# Pleasure or Health? The Role of Mental Simulation in Desire and Food Choices

**DOI:** 10.3390/foods9081099

**Published:** 2020-08-12

**Authors:** Naomí C. Muñoz-Vilches, Hans C. M. van Trijp, Betina Piqueras-Fiszman

**Affiliations:** Marketing and Consumer Behaviour Group, Wageningen University & Research, Hollandseweg 1, 6706 KN Wageningen, The Netherlands; hans.vantrijp@wur.nl (H.C.M.v.T.); betina.piquerasfiszman@wur.nl (B.P.-F.)

**Keywords:** desire, healthy choice, mental simulation, grounded cognition, imagery, implicit motivation, visual attention

## Abstract

Many times, desire possesses us and impedes us from making healthier food choices. From a grounded cognition perspective, we investigated the role of two types of mental simulation (process and outcome) in desire and food choice to understand the processes that modulate them and find strategies that encourage healthier food choices. In addition to these explicit measures, we used two implicit methods to measure approach-avoidance tendencies and visual attention. Our results showed that imagining the consumption of vice and virtue foods increased desire for the product imagined and seemed to favor the choice of a vice food. However, at an implicit level, the motivation to approach and avoid food products was neutral. Imagining the post-consumption of a vice food decreased desire for the imagined food and although it tempted people at an implicit level, it made people more prone to choose a virtue food. When a vice food was imagined, attentional bias increased for all types of food regardless of the simulation. When a virtue food was imagined, there was no effect on choice, motivation nor attentional bias. In conclusion, simply imagining certain foods is a potential solution for promoting healthier and thoughtful choices.

## 1. Introduction

Understanding desire has become essential in a world of temptations. Desire is defined as *“a psychological state of motivation towards a specific stimulus or experience that is anticipated to be rewarding”* [1]. Complementing the previous definition, we conceptualize desire as a fast/short-term reaction that is triggered by either an external cue (e.g., image) or that arises purely due to internal cognitive processes. An example of desire triggered by an external cue could be the smell of coffee when one is passing a café. However, what if you are just walking in a park and you suddenly have this desire for coffee? Many cognitive processes could have triggered it. One of them is, for instance, spontaneously remembering drinking coffee sitting on a bench of the forest with your friends. The Grounded Theory of Desire and Motivated Behavior [2] explains, by integrating psychological and neural mechanisms, why these situational experiences may trigger desire, and in turn affect behavior.

One central construct of the Grounded Theory of Desire and Motivated Behavior is situated conceptualization. Situated conceptualizations refer to all sensory features, contextual features, motor actions, current states, emotions, self-attributions, and much other information, which are all stored in memory, and are likely to be recreated or reenacted when one remembers an experience (situated conceptualization; [3,4]). For example, when one smells the scent of coffee, one could suddenly create simulations that recreate a highly pleasurable (or unpleasant) scenario of drinking coffee, such a scenario could be very rich in contextual, sensory features and emotions. Having vividly simulated the experience of drinking coffee could create instant desire (or rejection) for drinking coffee.

In normal circumstances, the situated conceptualizations, that may or not trigger desire arise rather spontaneously, without any conscious effort or even awareness, however, one could voluntarily simulate a certain experience which would create desire too (this research). In addition, the level of detail of those conceptualizations depends on the nature of the food. In a study [5], participants were asked to list features of four tempting/vice foods (e.g., chips) and four “neutral”/virtue foods (e.g., rice, vegetables). Participants used more words to describe the experience for tempting foods than for neutral foods, and they were more likely to describe a situated eating experience when they reported that the food was very attractive and elicited a strong desire to eat. Hence, more attractive/vice foods (or cues thereof) spontaneously activate richer situated conceptualizations than more virtue foods.

If we normally use simulations to predict desire for food (most often without our awareness), why do we not try to consciously guide simulated experiences to control desire and behavior? One way to make these simulations conscious is to instruct people to imagine their experience with a product. By instructing people to simulate an experience with a product, a situated conceptualization is activated, which reproduces many aspects of an earlier experience, including associated internal bodily and affective states. Although mental simulations are not a veridical representation of an object/food, and indeed, they are defused and lack so many details compared to a real experience, they serve as an approximation to the reality that helps the brain to minimize errors by bidirectionally giving feedback from cortical processes [6], which help to predict or anticipate value, either pleasure or health.

Attention modulates to some extent food choice [7], and additionally, is associated with obesity and food craving [8]. Attentional bias is a form of cognitive bias and refers to a preferential involvement of attention to one particular type of information (e.g., attractive and tempting food) [9]. Although this cognitively biased processing tends to be more automatically activated, it can be trained too. Several studies show the effectiveness at an interventional level [10]. Research on attentional bias training supports the idea that attentional bias for food impacts desire for food, food choices and food intake [11], for example, participants who were trained to attend chocolate pictures (visual attention) ate more chocolate in a taste test than people who had to attend to non-food stimuli [12]. The attentional bias modification paradigm can also encourage healthy eating by training the attentional focus toward healthier alternatives. In a study, participants who were trained to direct their attention towards pictures of healthy food had an attentional bias for such cues and ate more healthy than unhealthy snacks compared to the those trained to attend unhealthy food [13]. We, therefore, investigate whether process and outcome simulations can direct attention towards a particular food, and although they do not involve an explicit training as the attentional bias modification paradigm, we propose that reenacting a food (post)consumption event by simulating it in our mind would induce a certain mindset, which would shift visual attention towards information more aligned with the current mindset (i.e., health and indulgent mindset).

Considering the increasing obesity prevalence and a constant tempting environment, our aim is to investigate how mental simulation can promote the choice of virtue foods. The role of mental simulations in healthier behavior has gained interest in the past years, and indeed, a considerable amount of literature has been published on how eating simulations affect behavior [14,15,16,17,18]. However, the majority of the studies have only used or evoked consumption simulations (process simulations) but the use of post-consumption simulations (outcome simulations), especially in combination with virtue foods, has been poorly studied. For this purpose, we consider it to be of utmost importance to investigate as well the effects of simulating a post-consumption experience and of using virtue foods, since they are known to trigger different types of reward. Recent studies comparing process and outcome simulations have established that these simulations are a potential strategy to direct people into a healthier behavior since they can control desire and food choices [14,15]; however, the mechanisms underlying these relationships have yet to be further investigated.

Consumers are increasingly looking for healthier products, so companies have had to adapt and find strategies to satisfy consumers’ demand. However, products perceived as healthy (virtue) do not benefit from the same promotion strategies as more tempting products (vice). In fact, a recent study showed that evoking a multisensory experience of the food (e.g., taste, smell, enjoyment), a wide strategy used to promote vice foods [19] compared to a single-sense ad slogan (mentioning taste only), resulted in more negative thoughts when a healthy food (cherry tomatoes) was used [20]. Understanding how these simulations operate, also with virtue foods, is relevant to promote healthier choices at an individual level but it also contributes to a better understanding of how these foods behave in people’s minds, which could help companies to adapt their promotion strategies according to their product categories.

Hence, the type of food used to imagine (vice versus virtue) and the type of mental simulation (process versus outcome) seem to matter to be able to draw generalizable conclusions and therefore design appropriate strategies. However, these effects have been only reflected in rationalized responses. We, therefore, consider relevant to study this at an implicit level, to explore the link between automatic and rationalized motivational responses.

### 1.1. Theoretical Framework

The present research intends to confirm that mentally simulating the process of eating a vice food leads to a choice of vice foods, because of increased desire for the imagined food, whereas mentally simulating the outcome of having eaten a virtue food leads to a healthier choice because of increased desire for the imagined food. Importantly, we implicitly assess motivation to eat specifically vice or virtue foods under each simulation, and finally, we investigate whether these two simulations direct visual attention automatically towards a specific food category.

#### 1.1.1. Desire/Motivation and Food Choice

The Grounded Theory of Desire and Motivated Behavior [21,22] adds to the Elaborated Intrusion Theory of desire [23,24] that desire and its processes can be unconscious and conscious. In both theories, simulations are included as an important part of our everyday life desires. However, the EI theory [25] conceptualizes simulations as a top-down process, emerging spontaneously and intrusively from one’s mind (e.g., craving a food), and without the one’ awareness [26], these are named spontaneous mental simulations [27,28]. Although both theories place simulations, generally, in a nonconscious state and focus on top-down processes, we believe that we can use this brain capacity to evoke these simulations consciously and voluntarily. Indeed, studies, where consumption simulations of vice foods have been consciously evoked, have shown that appetitive reactions increased. For example, instructing people to imagine the consumption of food (process simulation) increased salivation compared to when participants simply looked at food pictures [29], and also increased expected enjoyment of smaller portions, which resulted in a smaller portion size selection [18]. Another example is, if one vividly imagines the smell of a vice food, salivation, desire and subsequent actual consumption of the food they imagine smelling will be greater, compared to when they do not imagine the smell [30]. Similarly, imagining eating a vice food increases salivation compared to a neutral food such as bread [29]. Some other studies have examined the effect of mental simulations on selections of portion sizes [16,18,31].

In this research, people simulate voluntarily and consciously. However, we believe that motivation to eat and attentional bias towards food occur implicitly and should be observable with measures that reflect a more automatic reaction rather than a rational one. Thus, by using implicit measures we intend to investigate whether the effects caused by mental simulations in desire and choice between a vice and a virtue product [15] are reflected at an implicit level.

Research in implicit approach-avoidance tendencies has established that motivational states and specific motor actions (i.e., arm flexion and extension) are directly linked. For example, a study showed that avoidance movements (compared to approach ones) and approach movements towards positive appraised foods (compared to negative ones) were faster for non-hungry participants (using a joystick). Moreover, this study showed that for negative/disgusting foods, the motivational tendencies were reflected in the implicit measures but not in the explicit ratings of pleasantness and wanting as the positive and neutral images showed [32], highlighting the importance of measuring implicitly. Hence, it is relevant to know whether the effect of process and outcome simulation on the desire for the imagined food and choice between a vice and virtue food is linked to short-term/implicit reactions.

#### 1.1.2. Attention

It has been suggested that a determinant of choice is the attention that is assigned to certain attributes of food [7,33], and that attention can be manipulated through priming [34] or induction of mindsets [35]. A functional magnetic resonance imaging (fMRI) study showed that non-dieters made healthier choices when the attentional focus was directed to the health aspects of food, suggesting that mental simulations (comparable to the studies in mindset induction), specifically outcome simulation, may lead to healthier decisions because an individual’s attention is directed towards longer-term features. In the same vein, another fMRI study, in which attention was manipulated through induction of mindsets (e.g., indulgent mindset, health mindset, fullness mindset), showed that, compared to a free choice condition, the selection of portion size with the fullness mindset was bigger due to increased activity in the insula, responsible for integrative interoceptive processes such as satiation. However, in the case of pleasure and health mindset, the selected portion size was smaller. Both mindsets were associated with their neural correlates. In the health mindset, there was an activation of the region of the brain which exerts cognitive control, i.e., prefrontal cortex, specifically DLPFC (dorsolateral prefrontal cortex), which is in charge of self-control; while in the pleasure mindset an activation of the OFC (orbitofrontal cortex) area was observed; this area is associated with the subjective pleasantness of food [36]. Moreover, in another study, a healthy versus an indulgent mindset was induced. The health mindset was induced by asking people to select food from a menu, consisting in four low-calorie foods, for their best friend who wants to lose weight, whereas the indulgent mindset was induced by asking people to select a food from a menu, consisting in four high-calorie foods, for their best friend who is getting married. They found that attention (measured with a visual probe task) towards high-calorie foods was attenuated only in participants who scored high in the level of eating restraint [37]. Similar research has shown that a health prime resulted in longer fixation time on low energy foods, also increased low-calorie food choices, and decreased high-calorie food choices [34].

From this evidence that is simply asking people to consider health versus indulgent aspects of foods can actually activate their neural correlates, we expect that mentally simulating an experience (either focused on the process or the outcome of eating a food product), which is rich in situated conceptualizations, will actually have a similar effect. Therefore, we explore whether mental simulations cause a certain state of motivation and whether it causes an attentional bias towards a specific food category (vice or virtue). Outcome simulation would automatically draw visual attention toward health-related aspects (virtue food pictures) since people would enter into a healthier mindset. On the other hand, we expect that process simulation will work in the same direction and will draw visual attention automatically towards information matching with their current motivational state (e.g., indulgent mindset), that is, vice food images. Last but not least, the changes in attention and motivation would have an effect on desire and choice between a vice and virtue food, with process simulation favoring vice food choices, and outcome simulation favoring virtue food choices.

## 2. Materials and Methods

### 2.1. Participants

Eighty students from Wageningen University and Research participated in this study (73.8% were female and 26.3% were male), the average age was 23.63 years old and the BMI was 21.5 kg/m^2^. They were recruited via posters, social media, and advertisements. The inclusion criteria were not being vegan, vegetarian, lactose intolerant, or having any food allergy, and refraining from eating at least 2 h before participation. The study was approved by the ethical committee of the Social Sciences School of Wageningen University and Research. As compensation, participants received a EUR 5 gift card for various stores in the Netherlands and a snack.

### 2.2. Procedure

Upon arrival, all participants provided written informed consent. Before any mental simulation, participants rated their correspondent food product for desire, expected enjoyment, and hunger, to determine their baseline measurements (see Table 1). The food products selected were banana (virtue) and chocolate cookies (vice). They were then asked to follow a bogus “training” of a dot-probe task (DPT) to capture initial attentional biases. Once they had finished the “training”, participants were randomly assigned to perform process simulation of either the vice or the virtue product, or outcome simulation of the same foods as in process simulation, resulting in four experimental groups. After the mental simulation phase, they rated the levels of desire for the imagined food, expected enjoyment, and hunger. Subsequently, they performed again a DPT to assesses automatic attention toward different food categories. At the end of the session, participants performed an Approach-Avoidance Procedure (AAP) to measure the approach-avoidance tendencies towards food, and participants were asked to choose between two products representing a virtue (grapes) and a vice (chocolate bar) dimension. Participants thought that they were going to consume these products during the session, but they did not. As a compensation, they could choose a snack at the very end of the study. After the choice task, participants filled in the Dutch Eating Behavior Questionnaire (DEBQ; [38]) to assess the level of restrained eating, emotional eating, and external eating to control possible differences between groups.

#### 2.2.1. Approach-Avoidance Procedure (AAP)

We followed the same procedure as other study [32]. Before the task started, participants were asked to feel comfortable while holding the joystick (Logitech, Extreme, Switzerland) and to try all the possible movements. They were instructed that when a grey triangle appeared in the upper part of the image they had to move the joystick forward and when the triangle appeared in the lower part of the picture the movement had to be backward (push or pull, respectively). They started with a practice which contained only neutral images (office tools). A trial started with the appearance of a fixation cross in the center of the screen. After 2000 ms, the cross disappeared and was replaced with the stimulus image in the center of the screen. The stimulus image disappeared when they pulled or pushed the joystick, and a new fixation cross and stimulus appeared.

#### 2.2.2. Dot Probe Task (DPT)

**Figure 1:**
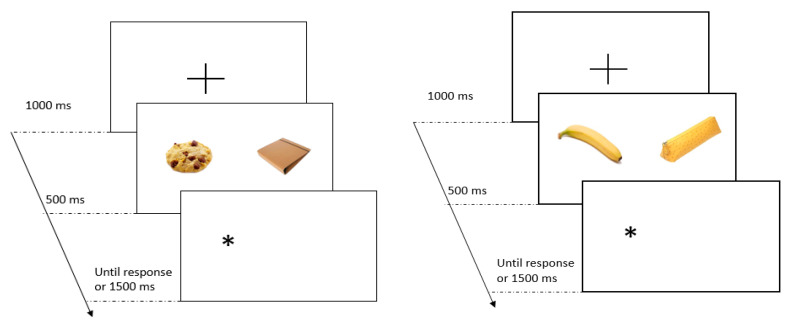
Two illustrations of the dot-probe task, the left with a vice product and the right with a virtue product. After participants were presented with the fixation cross, they were shown two pictures simultaneously for 500 ms, one corresponding to a food (healthy or unhealthy), and the other corresponding to a neutral pair. Finally, they were asked to press “B” if the asterisk appeared on the left side and “N” if the asterisk appeared on the right side.

Allocation of attention can be measured indirectly with a DPT using response latencies [39]. The DPT consists in presenting two images (a food and a non-food picture), one on the left and other on the right side of the screen, followed by the appearance of a dot (here, an asterisk) on one of the sides, right after the picture pair disappeared (see Figure 1). The logic behind this task is that the faster the participant presses the key where the dot is, the more likely they are to be attending to the image that was in that position.

The visual DPT included 80 trials (plus 8 practice trials with unrelated stimuli). The trials consisted of 20 stimuli pairs of food and a non-food pictures (see Figure 1). Each picture of the pair was equally presented on the left and the right side of the screen. The pairs were presented randomly four times (all the possible combinations regarding the position of the asterisk and food image).

### 2.3. Apparatus and Materials

The software used for the DPT was OpenSesame 3.2.4 [40]. For the AAP, the software used was E-Prime 2.0 (Psychology Software Tools, Pittsburgh, PA, USA) [41], and it was performed after the second DPT. Participants conducted the AAP task on a separate computer.

### 2.4. Stimuli

#### 2.4.1. Approach-Avoidance Procedure (AAP)

This method has been used to investigate the motivational approach and avoidance responses towards food. The logic behind the task is that *“people spontaneously approach positive evaluated, attractive stimuli and avoid negatively appraised, aversive stimuli”* [32].

**Figure 2:**
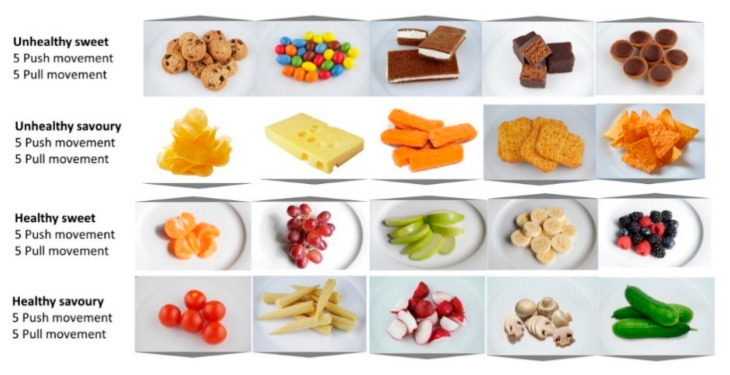
Examples of target images from the stimuli set used in the Approach-Avoidance Procedure.

The images were classified as being either “healthy” and “unhealthy” (See Figure 2). The healthy category contained 5 pictures of fruits and 5 of vegetables, while the unhealthy category contained 5 sweet high-caloric processed foods and 5 savory high-caloric processed foods. These images were taken from two picture sets [32,42], the images of the “healthy sweet” category were taken for this study. Each picture was presented two times, once with the triangle pointing upwards and once with the triangle pointing downward. Thus, each participant completed a total of 40 trials.

#### 2.4.2. Dot Probe Task (DPT)

The stimuli consisted of a set of target food photographs that were classified as being either “healthy” and “unhealthy”. These pictures were taken from the food-pics database [42]. Of the food pictures, 10 were healthy food pictures, and 10 unhealthy food pictures, each food picture was paired with a non-food picture (neutral). All image pairs were matched as closely as possible concerning color, complexity, brightness, and size.

### 2.5. Design and Data Analyses

The design for the explicit measures was 2 (mental simulation: process and outcome) × 2 (products imagined: banana and chocolate cookies) × 2 (time: before and after), except for the choice analysis that did not consider time. The factors “mental simulation type” and “products imagined” were between-subject factors, and “time” was a within-subjects factor. The design for the DPT task was the same as the explicit measures but with an additional within-subjects factor called “picture category” (healthy and unhealthy). The AAP design was the same as the DPT but without the factor “time”.

The three tasks followed the same analysis. Thus, two independent repeated-measures ANOVAs were conducted, one to test the effect of simulating the process and the outcome of eating a vice product (chocolate cookies), and another to test the effect of simulating the process and the outcome of eating a virtue product (banana).

#### 2.5.1. Approach-Avoidance Procedure (AAP)

The dependent variable was the approach-avoidance index (AA-index), which was calculated by subtracting the approach reaction times from the avoidance reaction times (RT_avoidance_-RT_approach_). The aim was to test the approach and avoidance movements relatively and to see whether these movements differed from each other among the mental simulation types (process versus outcome) when the target images of different (un-) healthy food categories appeared on the screen. All incorrect responses were excluded from the analysis.

#### 2.5.2. Dot Probe Task (DPT)

The dependent variable was attentional bias (ms) towards food pictures. From the design, we have four measures of attentional bias per product imagined (chocolate cookies and banana). Four corresponding to the attentional bias for the healthy food images (before and after simulation) in process simulation, and four corresponding to the attentional bias for the unhealthy food images (before and after simulation) in outcome simulation. These variables were created by subtracting the reaction time (ms) of the healthy or unhealthy images to the reaction time (ms) of its neutral pair. Therefore, a positive result would mean that participants were faster in reacting when the asterisk was in the healthy food picture (attentional bias towards food). The same applies to unhealthy food pictures. In total the data of three participants were removed, one because their accuracy was below 50%, and two others for technical problems that impeded us to record a part of the data. We also removed the incorrect trials and outliers (−3SD, +3SD over the mean of each variable).

## 3. Results

### 3.1. Baseline Measures

Regarding baseline measures, the groups did not differ in terms of desire (F(376) = 0.182, *p* = 0.908), expected enjoyment (F(376) = 0.548, *p* = 0.651), and hunger (F(376) = 0.349, *p* = 0.790). Moreover, differences in personal traits (restrained, emotional and external eaters) between the four groups were checked for. There were no significant differences between the mental simulation types, when vice and virtue product were imagined, for restrained eating (F(379) = 0.555, *p* = 0.646), emotional eating (F(379) = 1.787, *p* = 0.157), and external eating (F(379) = 0.627, *p* = 0.600).

### 3.2. Effect of Mental Simulation on Desire

#### 3.2.1. Effect of Mentally Simulating the Vice Food (Chocolate Cookies)

Regarding the results for desire, “time” as the main effect was marginally significant (F(140) = 3.10, *p* = 0.086). We found no main effect for the factor “mental simulation type” F(140) = 1.68, *p* = 0.203, but a marginally significant interaction between “time” and “mental simulation type” F(140) = 3.84, *p* = 0.057. Process simulation increased the desire for the vice imagined food while outcome simulation had no effect.

#### 3.2.2. Effect of Mentally Simulating the Virtue Food (Banana)

Regarding the desire for the virtue food, results show that there was a main effect of “time” for the virtue product (F(136) = 7.67, *p* = 0.009). We found no main effect of the “mental simulation type” or its interaction with “time”, both mental simulations increased desire for the imagined virtue food.

### 3.3. Other Measures

Since it has been suggested that multisensory imagery (equivalent to process simulation) affect portion sizes by diminishing the effect of otherwise salient feelings, such as hunger [16,18], we considered it relevant to add expected enjoyment and hunger for a manipulation check. The results of expected enjoyment showed that “time” was a significant factor (F(176) = 10.75, *p* = 0.002). In process and outcome simulation, expected enjoyment significantly increased but we found no significant effects on the interaction “time” and “mental simulation type” (F(176) = 0.911, *p* = 0.440). Regarding hunger, the results showed that “time” was a significant factor (F(176) = 6.69, *p* = 0.012); after the mental simulation the levels of hunger increased, especially after process simulation (M_baseline_ = 4.5, M_process_ = 5.2). Although the interaction between “mental simulation type” and “time” was not significant (F(176) = 1.02, *p* = 0.387), pairwise comparisons showed that hunger significantly increased after having simulated the process of eating the vice food (*p* = 0.007), and there was no effect of outcome simulation on hunger.

### 3.4. Effect of Mental Simulation on Choice between Vice and Virtue Foods

Two different Chi-square tests were conducted to analyze to what extent mental simulation had an impact on people’s choices between a virtue and vice product in an independent task (grapes versus a chocolate bar). We conducted one chi-square for the group who imagined the vice product (chocolate cookies), and another one for the people who imagined the virtue product (banana). Product choice frequency was our dependent variable, and simulation type our independent variable.

The results presented in Table 2 show that process simulation and outcome simulation had a significant effect on the probability of choice for people who imagined the vice product, *χ^2^* (1) = 4.624, *p* = 0.032. Mentally simulating the process of eating a vice product (chocolate cookies) led to a higher proportion of people choosing the vice product (chocolate bar) compared to imagining the outcome of such consumption (65% vs. 31.8%). However, imagining having eaten the vice product (chocolate cookies) led to a higher proportion of people choosing the virtue product (grapes) compared to process simulation (68.3% vs. 35%, respectively). For people who imagined the process and outcome of eating the virtue product (banana), although we found the same pattern, there were no significant differences between process and outcome simulation *χ^2^* (1) = 0.920, *p* = 0.338.

### 3.5. Effect of Mental Simulation on Implicit Motivation (AAP)

#### 3.5.1. Effect of Mentally Simulating the Vice Food (Chocolate Cookies)

**Figure 3:**
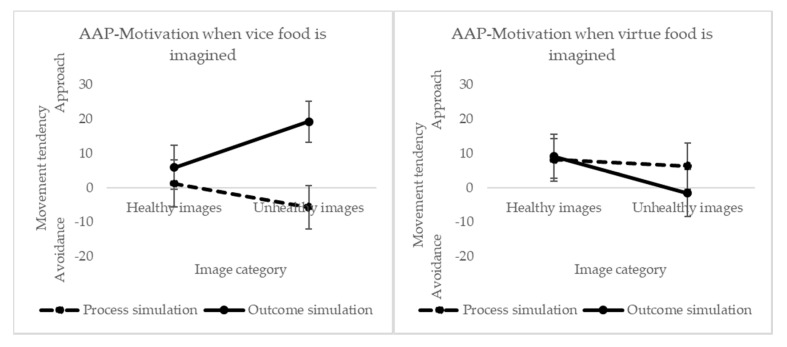
Approach-avoidance tendencies by type of mental simulation and product imagined.

Results show a marginal two-way interaction effect between “picture category” (healthy versus unhealthy stimuli) and mental “simulation type” (F(1414) = 2.72, *p* = 0.100). Pairwise comparisons show that approaching unhealthy images in the outcome simulation is significantly higher than in the process simulation (M_process_ = −5.74, M_outcome_ = 19.29, *p* = 0.004). We found no effect of picture category (F(1414) = 2.26, *p* = 0.607), but a significant effect of mental simulation type (F(1414) = 5.12, *p* = 0.024). These results show that imagining the outcome of having eaten a vice food led to faster approach movements towards unhealthy food stimuli (see Figure 3). At the same time, unexpectedly, mentally simulating the process of eating a vice food led to faster avoidance movements towards unhealthy foods compared to outcome simulation.

#### 3.5.2. Effect of Mentally Simulating the Virtue Food (Banana)

There was no significant effect on any of the factors nor interactions. We see in Figure 3 that, for the healthy food images, both types of mental simulations led to a similar approach tendency. However, for the unhealthier food images, we observe that outcome simulation led to a neutral tendency, and that process simulation led to an approach tendency.

### 3.6. Effect of Mental Simulation on Attentional Bias (DPT)

#### 3.6.1. Effect of Mentally Simulating the Vice Food (Chocolate Cookies)

**Figure 4:**
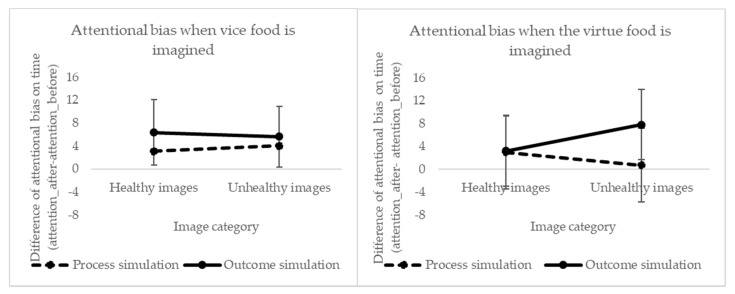
Difference between before and after simulation, when people imagined the vice and the virtue food product, across the picture food category.

The three-way interaction between “time”, “picture category” and “mental simulation type” was not significant (F(1731) = 0.015, *p* = 0.903) (see Figure 4). We found a significant interaction between “picture category” and “mental simulation type” (F(1731) = 4.425, *p* = 0.036). There was also a significant main effect of “picture category” (F(1731) = 12.743 *p* < 0.001), meaning that people had a natural attentional bias towards more healthy products, regardless of the type of mental simulation and time (M_healthy_ = 13.59, SD_healthy_ = 2.04, M_unhealthy_ = 3.52, SD_unhealthy_ = 2.07). There was a marginally significant difference in the factor “time” (F(1731) = 2.797, *p* = 0.095), meaning that regardless of the picture category and mental simulation type, people had a positive bias towards food after simulation.

#### 3.6.2. Effect of Mentally Simulating the Virtue Food (Banana)

The factor “picture category” was significant (F(1665) = 6.2, *p* = 0.013) (see Figure 4), meaning that people had an attentional bias towards more healthy foods, regardless of the mental simulation type and time (M_healthy_ = 11.67, SD_healthy_ = 2.30, Mu_nhealthy_ = 3.35, SD_unhealthy_ = 2.32). The results show no significant differences in time (F(1732) = 0.47, *p* = 0.495) nor any of its interactions, meaning that having imagined any of the mental simulation types did not affect the attentional focus of participants. No significant interactions between the other factors were found, thus they will not be further discussed.

### 3.7. Summary of the Results

In order to give a clear overview and simplify the findings of the implicit and explicit measures, we elaborate the results in Table 3.

## 4. Discussion

This research intended to investigate how two different types of mental simulation (process versus outcome), using as imagination object a vice or a virtue food, impacts desire for the imagined food and food choice between a virtue and vice snack. Moreover, it examines the effect that these two mental simulations have on visual attention that is automatically directed towards a certain food category (healthy versus unhealthy) and implicit motivation and explores their relationship with desire and choice.

Regarding imagining the vice food, our results are in line with the theories based on grounded cognition [2,23], and with previous studies where imagining the process of eating a vice food increased desire for the food imagined and also favored a vice food choice, whereas imagining the outcome of having eaten a vice food decreased desire for the food imagined and made people more prone to choose a virtue food [15]. Extending previous research [15], we found that people not only choose the food that is “matching” with the simulation [14] but in this research, we showed that this also happens for another product in the same vice/virtue category. This is in line with previous studies suggesting that exposure to food cues (e.g., smell) implicitly increases appetite for foods with similar properties [43,44,45,46]. For example, people choose more fruity desserts when they are primed with a pear odor, compared to people who are not primed with any odor, who choose more frequently a brownie option [44]. Hence, imagining a certain experience would automatically activate associated representations in memory, which would be more accessible at the moment of choice.

With this research, we contribute to the understanding of mental processes considering more neutral/healthy foods. However, our findings show that imagining a virtue product does not have a big impact on the explicit measures of desire and food choices, or on the implicit measures of motivation and attentional bias. Since imagining the virtue food provoked a higher desire in the process and outcome simulation, both simulations likely created positive thoughts, just as when a malleable food is imagined (contains hedonic and utilitarian characteristics; [14]). The fact that mental simulation was not very effective with virtue foods could be because virtue foods evoke fewer feelings of guilt or temptation thoughts [47] while vice foods tend to elicit more negative thoughts about the consequences of eating, which in turn influence choice for a virtue/healthier alternative. In other words, the extent to which a simulation affects desire depends on the valence of the content and its associations, and therefore the nature of the food category used for the simulation.

Therefore, if the valence modulates the effect of the desire for the imagined food one would expect to see this reflected implicitly, since implicit methods are often used to capture affective reactions, which are normally considered to reflect relatively fast processes, and indeed precede the cognitive and deliberate reactions [48]. Yet, unexpectantly, imagining the consumption of a vice food (process simulation) resulted in an avoidance tendency towards vice foods. This pattern of results could be explained by an asymmetry in the association between goals and temptations ([49]; Study 1). This means that process simulation (called temptation prime in their original study) would actually activate a health goal, showed by relatively avoiding faster than approaching unhealthy foods in this simulation type. On the other hand, imagining the post-consumption (outcome simulation) of the vice food led to an approach tendency towards vice foods, meaning that vice foods inevitably tempted people (as first impression) but when people rationalized the consequences, they were more able to choose a food product according to their higher-order beliefs (having a healthy diet). This pattern fits with previous research that has shown that humans process taste-related information faster than health-related information [50], and automatically detect hedonic food information such as tastiness but not healthfulness [51], which fits with the idea that taste corresponds to a more visceral and short-term basis; while the concept of healthfulness is a higher-level construct, which is commonly conceived to be achievable further in the future. Process simulation likely has a more intuitive and fast nature, whereas outcome simulation a more rational and reflective one. This notion is aligned with dual-process theories, which acknowledge the existence of an intuitive, fast, automatic system; and a deliberate, reflective, and rule-based system [52]. Hence, results showed that people were tempted implicitly towards vice foods when imagining the post-consumption of a vice food, but they did not express it in the food choice task after having the time to think about it for longer, which could mean that the mechanism by which outcome simulation encourages healthier choices has a rational root rather than an intuitive one. However, this explanation remains rather speculative and further investigation into this result is warranted.

Last but not least, as attention is directed automatically towards those stimuli that are relevant given the current mindset [53], we expected that imagining consuming or having consumed a vice food would direct attention towards vice and virtue food images, respectively. With the current experimental setup, we did not observe these results. The results show that there was an attentional bias towards all types of foods indistinguishably. One could wonder whether this increased attention for all type of foods was due to a learning effect between the two sessions, similar to the principle of attentional bias modification [13,54,55] but we think that this was not the case, since we only observed it when people imagined the vice food. When the virtue food was imagined, there was no effect of attentional bias. At this point, it is worth noticing that this research did not use a particular population (e.g., restrained eaters), thus it might be that the majority of our participants did not feel a conflict between following a diet and indulgent eating, which could have diminished the effect of implicit motivation and attentional bias. Indeed, research has shown that the extent that motivational state (i.e., being in indulgent mindset versus a health mindset) affects attentional bias depends on individual differences, such as the level of eating restraint [38,56]. In Werthmann and colleagues’ experiment, only the group with higher restrained eating showed differences in response latency bias when they were induced in a healthy and indulgent mindset. Only high restrained eaters paid more attention to high-calorie food in the indulgent mindset and less attention to these foods in a health mindset. This may explain why visual attention was automatically directed towards all types of foods, when the vice food was imagined, in an average population, and that there was no effect when the virtue food is simulated. Hence, we could say that process and outcome simulation increased the salience of food items when the vice food was simulated. These results are in line with research showing that the rewarding effect of eating a liked food (or imagining eating in this research) can cause the cues related with food (e.g., sight, smell) to gain incentive salience, provoking a potential to attract attention (for a review see Werthmann, Jansen, et al., 2014). Furthermore, other studies have shown that attention and craving are closely related [57,58]. Even imagining food consumption versus a non-food scenario (having holidays) increases the intensity of craving, especially for dieters [59], which is likely to affect attentional bias for food.

Taken together and linking these findings to those of desire and choice, we can conclude that the consequences of mental simulation in automatic motivational and attentional responses are slightly different compared to those where individuals have some time to reflect on their decisions (ratings of desire and choice in our study). A direct comparison was not intended, but rather we sought to explore the relationships between the two outcomes, since to the best of our knowledge, this is the first study investigating the impact that two different types of mental simulations have on implicit motivation and attentional bias in the food context.

### Future Research and Implications

The most important implication is that imagining the process versus the consequences of having eaten a vice food impacts food choice between a vice and a virtue food (different than the imagined food). Moreover, contrary to our expectations, we found that outcome simulation tempted people at an implicit level, but this effect was not translated in a rational choice task. Therefore, future research could examine whether outcome simulation is modulated by higher-order processes and to what extent they are different from the ones that modulate the process simulation, which are more related to instant reward.

Many research findings suggest that restrained eaters differ from healthy individuals in terms of eating behavior. This study explored the effect of imagined food (post) consumption only on a healthy population. Future research could extend this research by investigating how these findings vary among people with eating disorders.

Some practical implications are that people could simulate the consequences of a vice food to help themselves to choose healthier snacks, companies could promote healthier foods evoking more outcome-like simulations, for instance, letting people know about the satiating properties of food, the energy needed for sport or about helping them to achieve the healthy lifestyle goal.

Finally, we also encounter some methodological limitations. This study had many steps, which could have affected our results. For example, the AAP was performed after the DPT task, thus, it is possible that this contributed to diminishing the effect of the AAP task. Yet, we do not expect that this changed the pattern of our results, since the choice task, which was performed at the very end, is very consistent across different studies [14,15].

## 5. Conclusions

In conclusion, mentally simulating the (post-) consumption of a vice versus a virtue food matters. Therefore, the understanding of mental simulations and their implications is key for the development of interventions and marketing communication strategies, which could encourage consumers to adopt healthier dietary patterns. Simply imagining food is a potential solution for generating healthier and thoughtful choices.

## Figures and Tables

**Table 1:** Variables involved in the experimental procedure in chronological order.

Baseline measures	Desire for the virtue and vice food Expected enjoyment for the virtue and vice food Hunger
Dot probe task (DPT) (framed as “training”)	Attentional bias towards vice food pictures Attentional bias towards virtue food pictures
Mental simulation	Process simulation (imagining the consumption) Outcome simulation (imagining the post-consumption)
Measures after simulation	Desire for the virtue and vice food Expected enjoyment for the virtue and vice food Hunger
Dot probe task (DPT)	Attentional bias towards vice food pictures Attentional bias towards virtue food pictures
Approach-Avoidance Procedure (AAP)	Relative approach vs. avoidance towards virtue and vice food pictures
Food choice	Virtue food vs. vice food
DEBQ	Restrained eating, emotional eating, and external eating.

**Table 2:** Choice frequency (%) of participants’ choices in each mental simulation, in which participants imagined a vice product (chocolate cookies) and a virtue product (banana). Different letters across columns represent significant differences between mental simulation type.

Product Category of the Simulation	Product Choice	Process	Outcome	Chi-Square
Imagining a vice product	Vice choice	65.0% a	31.8% b	*χ^2^* (1) = 4.624, *p* = 0.032
	Virtue choice	35.0% a	68.2% b	
Imagining a virtue product	Vice choice	60.0% a	44.4% a	*χ^2^* (1) = 0.920, *p* = 0.338
	Virtue choice	40.0% a	55.6% a	

**Table 3:** Summary of results.

Product Imagined	Vice Product (Chocolate Cookies)		Virtue Product (Banana)	
Mental Simulation	Process	Outcome	Process	Outcome
Desire for imagined food	Increase desire for imagined food	Decrease desire for imagined food	Increase desire for imagined food	Increase desire for imagined food
Choice between vice and virtue	Greater likelihood for vice choice	Greater likelihood for virtue choice	No significant but same trend as with imagined vice food	No significant but same trend as with imagined vice food
Implicit motivation (AAP)	Neutral motivation for healthy and unhealthy foods	Neutral motivation for healthy foods and high motivation for unhealthy food	Moderate motivation for healthy and unhealthy foods, although no significant	Moderate motivation for healthy and neutral motivation for unhealthy foods, although no significant
Attentional bias (DPT)	Greater attention towards all types of food	Greater attention towards all types of food	No effect	No effect
